# Onwards and upwards

**DOI:** 10.1093/jxb/eraf126

**Published:** 2025-07-02

**Authors:** John E Lunn

**Affiliations:** Max Planck Institute of Molecular Plant Physiology, D-14476 Potsdam-Golm, Germany

**Fig. 1.**
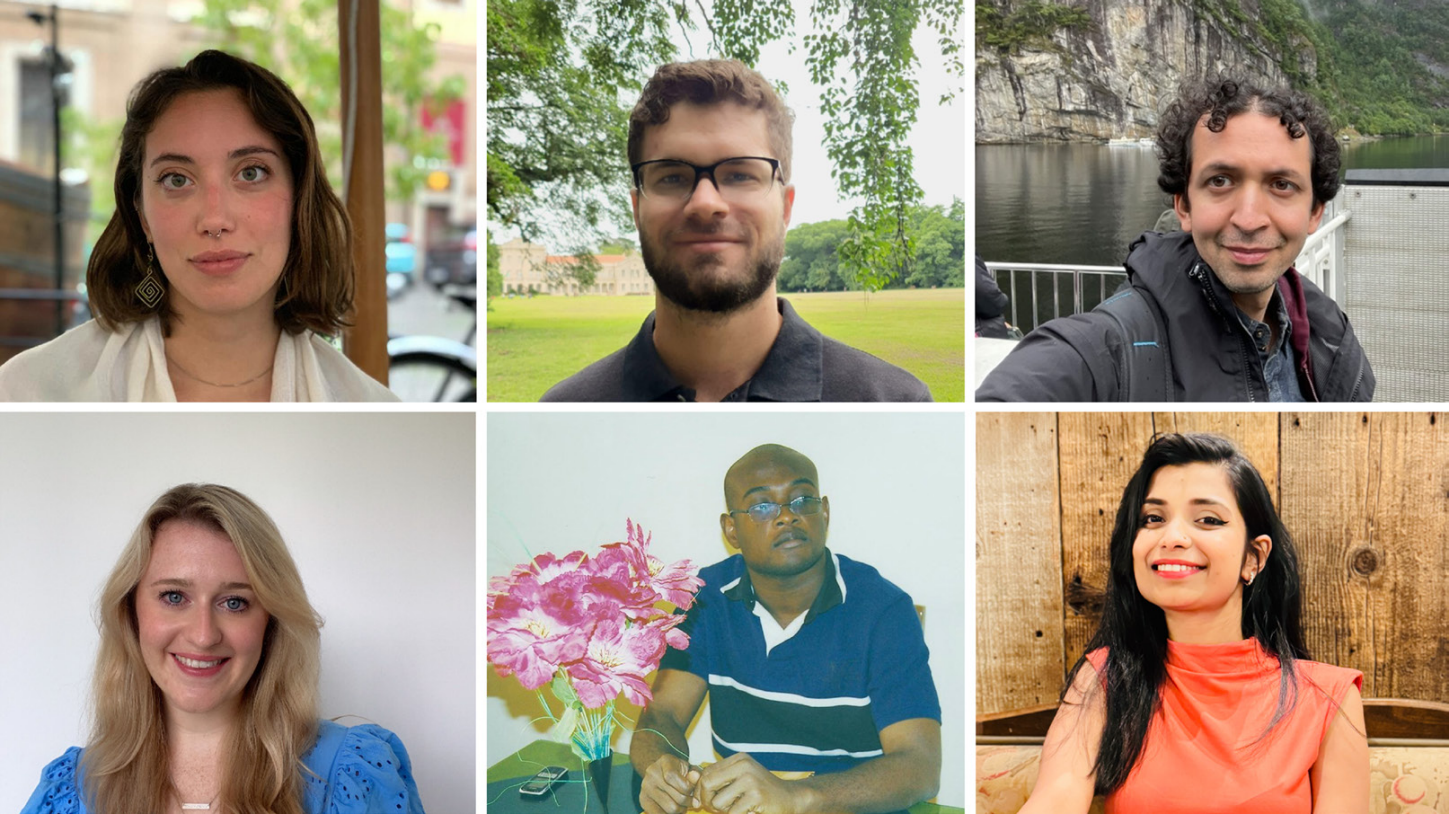
JXB Editorial Interns for 2025. Top row (L to R): Vittoria Clapero, Níkolas Mateus, Luis Alonso Baez. Bottom row (L to R): Katie Watson, Henry Njoku, Deeksha Singh.

**As we enter a new, and rather special, year for the *Journal of Experimental Botany*, we welcome a new cohort of editorial interns for 2025: Luis Alonso Baez (Norwegian University of Science & Technology, Norway), Vittoria Clapero (Max Planck Institute of Molecular Plant Physiology, Germany), Níkolas Mateus (University of São Paulo, Brazil), Henry Njoku (University of Ibadan, Nigeria), Deeksha Singh (University of California, Davis, USA), and Katie Watson (University of Hong Kong, Hong Kong) (Fig. 1). JXB editorial internships provide early career researchers with an opportunity to learn how a scientific journal works from the inside, opening up new horizons for their future careers.**

Each of our interns is mentored by two of our scientific editors as they learn about the editorial review process, from initial assessment through to publication. The usual rules of manuscript confidentiality apply, and the Handling Editor is solely responsible for the final decision on each manuscript. Our interns will also receive guidance for writing Insight articles to highlight selected papers published in JXB, and there will be opportunities to contribute to the journal in other ways, such as providing content for our social media channels. To learn more about our editorial interns, please visit our website: https://academic.oup.com/jxb/pages/editorial-interns. I would like to thank our 2024 editorial alumni—Diego Pinheiro Brito, Hannah Drieberg, Natalie Hoffmann, Sujit Jung Karki, Martin Mburu, and Tessa Reid—for their valuable contributions to the work of the journal over the last year, and to wish them success in their future careers. I would also like to express my grateful thanks to JXB editors Angus Murphy, Elspeth MacRae, and Richard Napier, who kindly gave up their time to serve as the selection committee.

As a society journal, fully owned and managed by the Society for Experimental Biology (SEB), our sole mission is to serve the science community. In turn, our community of authors, reviewers, and editors is supported by our Editorial Office team in Lancaster, UK, whose dedicated work behind the scenes is essential for the efficient and professional operation of the journal. In particular, I would like to mention Dr Raquel Gonzalez-Cuesta, who is retiring this year after more than 20 years of invaluable service to the journal. We will greatly miss Raquel, not only for her scientific knowledge and experience but also as a valued colleague and friend. We wish Raquel a long and happy retirement.

The peer review system is dependent on the knowledge, expertise, and goodwill of our reviewers, who help JXB’s editors to provide a rigorous assessment of manuscripts and constructive advice for our authors. In recognition of their valued contributions to the journal, we have invited some of our most active and experienced reviewers to join our new Editorial Advisory Board (https://academic.oup.com/jxb/pages/editorial-advisory-board). This panel of expert reviewers will help to maintain our rigorous peer review standards. With representatives from diverse research fields and different parts of the world, our Editorial Advisory Board will also act as a channel for feedback from the international plant science community. This will help us adapt to the challenges of the future, to ensure that JXB remains a trusted and respected journal for publication of exciting and impactful plant science.

As mentioned above, 2025 is a special year for JXB as we celebrate the 75th anniversary of the journal’s foundation by the SEB in 1950. To mark this historic milestone, while also looking ahead to the future, JXB and the SEB are organizing a scientific meeting in Edinburgh (17–19 September 2025) with a focus on early- and mid-career researchers. The meeting will bring together young, and some not so young, scientists from different fields to share their science, exchange ideas, and build new connections as we look ahead to the next 25 years. We will also be running workshops on scientific publishing, ethics, and careers. Details of the programme, venue, and registration are available here: https://www.sebiology.org/events/journal-of-experimental-botany-symposium.html. We look forward to welcoming many of you to this exciting conference and working on your behalf as we guide JXB onwards and upwards into the future.

